# Free Light Chain Monomer—Dimer Pattern Analysis as Non-Invasive Tool in Predicting MGUS and SMM Progression †

**DOI:** 10.3390/cancers18152409

**Published:** 2026-07-26

**Authors:** Avshalom Serok, Lesya (Olga) Kukuy, Omer Shaked, Omer Weinstein, Marjorie Pick, Amir Serok, Alina Ostrovsky, Rivka Goldis, Eyal Lebel, Batia Kaplan, Moshe E. Gatt

**Affiliations:** 1 Department of Internal Medicine, Hadassah Medical Center, The Hebrew University of Jerusalem, Jerusalem 91120, Israel; 2 Institute of Nephrology and Hypertension, Sheba Medical Center, Ramat Gan 52621, Israel; 3 Department of Hematology, Hadassah Medical Center, Faculty of Medicine, The Hebrew University of Jerusalem, Jerusalem 91120, Israelrmoshg@hadassah.org.il (M.E.G.); 4 The School of Computer Science and AI, Tel Aviv University, Tel Aviv 69978, Israel; 5 Institute of Hematology and Sheba Cancer Research Center, Sheba Medical Center, Ramat Gan 52621, Israelbatiakaplan@gmail.com (B.K.); 6 Department of Neurology, Sheba Medical Center, Ramat Gan 52621, Israel

**Keywords:** FLC, dimerization, MGUS, SMM, progression

## Abstract

**Background/Objectives**: MGUS and smoldering multiple myeloma (SMM) are precursor states of plasma cell disorders with variable risk of progression to multiple myeloma (MM). Yet, current risk stratification models combine clinical and laboratory parameters, including invasive bone marrow assessment, but have limited precision. **Methods**: We applied free light chain (FLC)-monomer (M)–dimer (D) pattern analysis (FLC-MDPA), a non-invasive Western blot–based serum assay, to detect abnormal FLC M–D patterns associated with early malignant transformation, for predicting progression in MGUS (*n* = 68) and SMM (*n* = 40). Among 96 patients with complete data, 50 formed a training set to define criteria for progressive disease and 46 comprised a validation set. **Results**: FLC-MDPA predicted biochemical progression (sensitivity 0.79, specificity 0.92, NPV 0.86; HR 19.96, 95% CI 4.31–92) and clinical progression (sensitivity 0.84, specificity 0.79; HR 13.5, 95% CI 3.68–49.64), with significantly higher progression rates in patients with abnormal patterns (*p* < 0.0001). Within this high-risk cohort, FLC-MDPA was associated with higher hazard ratios for both biochemical and clinical progression compared with the 2/20/20 model, while incorporation into a modified 2/20/MDPA model improved sensitivity and negative predictive value. In a multivariable model including MDPA and FLC ratio, MDPA was independently associated with both clinical (*p* = 0.001) and biochemical progression (*p* = 0.04). **Conclusions**: These findings suggest that FLC-MDPA is a promising non-invasive tool for risk stratification in MGUS and SMM, improving sensitivity and negative predictive value while potentially reducing reliance on bone marrow–based assessment.

## Simple Summary

Patients with monoclonal gammopathy of undetermined significance and smoldering multiple myeloma have precursor plasma cell disorders that may progress to active multiple myeloma. Predicting which patients are at high risk of progression is clinically important because early identification may improve monitoring and treatment decisions, but accurate risk assessment remains difficult. Current prediction models have limited precision and often rely on invasive bone marrow examinations. In our previous studies, we showed that abnormal free light chain monomer–dimer patterns were observed in multiple myeloma and light chain amyloidosis, but not in benign plasma cell disorders or healthy individuals. In this study, we aimed to evaluate whether testing for these abnormalities could help predict disease progression in pre-myeloma states. We found that abnormal free light chain patterns were strongly associated with progression and could improve current risk stratification models.

## 1. Introduction

Monoclonal gammopathy of undetermined significance (MGUS) and smoldering multiple myeloma (SMM) represent asymptomatic clonal plasma cell disorders (PCD) that can precede the development of multiple myeloma (MM). MGUS affects approximately 3% of individuals over the age of 50 and progresses to symptomatic disease at a modest rate of ~1% per year [1]. In contrast, SMM has a substantially higher risk of progression—estimated between 10% and 20% annually during the first five years. Identifying which patients will progress is of paramount importance, as early intervention in selected high-risk individuals may delay or even prevent the onset of end-organ damage [2] and allow early intervention treatment.

Current risk stratification models, such as the Mayo 2/20/20 model [3], integrate variables including M-protein concentration, bone marrow plasma cell (BMPC) percentage, and free light chain (FLC) ratio to estimate progression risk. Other risk models include bone marrow aberrant plasma cell (PC) content and presence of immunoglobulin immunoparesis [4], whereas older models focused on determination of heavy chain type and FLC ratio values [5]. While these models offer general prognostic guidance, their accuracy at the individual level remains suboptimal [6]. Patients within the same risk category often experience divergent clinical trajectories, and risk scores derived from cross-sectional measurements may not adequately reflect underlying disease biology [7]. Moreover, many high-risk patients remain under observation alone, as the clinical tools available today do not reliably distinguish indolent from biologically active disease [8]. This has prompted a growing interest in identifying new informative biomarkers that are particularly non-invasive and readily available for routine use [9,10]. Emerging evidence supporting early intervention in high-risk SMM (e.g., the AQUILA study [11]) further highlights the clinical importance of accurate risk stratification. Phase 3 data demonstrate that treatment with daratumumab is associated with a significantly reduced risk of progression compared to observation, underscoring the need for reliable tools to identify patients who may benefit from early intervention.

We have previously reported on a new non-invasive biomarker allowing differentiation of MM and light chain amyloidosis (AL) from asymptomatic PCD [12]. For this purpose, Western blotting (WB) was performed to analyze two major molecular forms of serum FLC, the monomers (M) and dimers (D). Covalent binding of FLC monomers into dimers involves an interchain disulfide bond between C-terminal FLC cysteines via oxidation of their thiol (SH) groups. This process is catalyzed by a family of oxireductases, while the activity of these enzymes depends on redox potential within different compartments of the cell. Under pathological conditions, such as oxidative stress, a shift in redox potential may occur, thus affecting the subtle balance between monomeric and dimeric states. Our previous studies demonstrated that pathological changes in FLC M–D patterns were observed in MM, AL and some SMM cases but not in MGUS or in healthy controls [12,13,14]. The observed abnormalities often included increased dimerization of FLC. This may be related to conformational changes in FLC molecules due to altered post-translational modifications and somatic mutations, or because of oxidative stress occurring under pathological conditions. Importantly, the applied FLC-M–D pattern analysis (FLC-MDPA) showed high sensitivity and negative predictive value for diagnosing AL from asymptomatic plasma cell disorders and healthy individuals. Also, this assay was capable of identifying subtle FLC M–D pattern abnormalities even in patients with minimal paraprotein burden [13,15]. Since we know that pre-malignant stages like SMM and MGUS are the biological precursors to MM, the fact that the same M–D patterns are detectable in both minimal and clear pathology suggests a continuous spectrum of clonal evolution. Thus, FLC-MDPA may assist in the detection of early malignant transformation, capturing a dynamic biological process that precedes measurable tumor burden or overt progression criteria.

In this study, we hypothesized that FLC-MDPA may serve not only as a diagnostic tool, but also help to predict disease progression to MM in the precursor states MGUS and SMM.

## 2. Materials and Methods

**Patients and Study Design.** This was a prospective, observational cohort study utilizing serum FLC-MDPA assay in patients with high risk MGUS or SMM, in correlation to progression. To ensure a representative clinical cohort and minimize selection bias, patients were enrolled consecutively during routine clinical follow-up at the Hematology Department of Hadassah Medical Center. Serum samples from patients with MGUS or SMM were obtained prospectively between 2014 and 2020 and the patients were monitored longitudinally.

Patients were classified as MGUS or SMM according to the diagnostic criteria of the International Myeloma Working Group (IMWG) [16]. From the total population screened during this period, 108 patients met the specific definition of relatively high-risk for progression, established biomarkers and mandated the presence of ≥1 risk factor. These included BMPC ≥ 20%, M-protein > 2 g/dL, FLC ratio > 8, non-IgG isotype, immunoparesis, and ≥95% aberrant clonal plasma cells by flow cytometry.

Among these 108 higher-risk patients, 96 had sufficient follow-up data for longitudinal progression analysis. The remaining 12 patients were excluded due to insufficient follow-up (2 deaths, 10 lost to follow-up). Survival analysis used the progression date for progressors and last follow-up date for non-progressors. To refine the FLC-MDPA criteria, this cohort was divided chronologically: the 50 earliest patients with the longest follow-up were utilized as a training set, while the subsequent 46 patients served as a validation set (Figure S1). Patients with overt multiple myeloma (MM), including SliM CRAB [16] at baseline or low-risk MGUS were excluded from the primary progression analyses cohort (*n* = 96) but were included and used as a reference control group to support the definition of MDPA criteria.

**Follow-up and Definitions of Biochemical and Clinical Progression.** Clinical progression was defined as the initiation of MM-specific therapy (Table S1) due to the development of CRAB criteria (hypercalcemia, renal insufficiency, anemia, bone lesions) or SLiM (60% PC, LC ratio, MRI lesions)-CRAB [16]. Biochemical progression was defined per IMWG criteria: a ≥25% increase in serum M-protein, with an absolute increase of more than 0.5 g/dL or involved/uninvolved FLC difference (dFLC) > 100 mg/L [17,18,19].

**Laboratory Analysis.** FLC-MDPA was performed as previously described [13,14], using the electrophoresis and blotting apparatus (Mini Vertical Electrophoresis System Xcell SureLock, Invitrogen, Carlsbad, CA, USA). Each electrophoretic run included a reference sample (a mixture of serum samples obtained from 15 healthy individuals) which was run alongside the tested serum samples. FLC κ and λ bands were immunodetected with rabbit antibodies to human Ig κ and λ light chains (DAKO, Carpinteria, CA, USA). The intensity of immunoreactive FLC bands (monomers and dimers) was quantified; to normalize the obtained values, the intensities of tested bands were divided by those of reference bands. The obtained relative levels of involved (i) and uninvolved (u) monomers (iM and uM) and dimers (iD and uD) were used to calculate the following FLC indices as previously described [14]:

(a) Dimerization index: iD/iM,
(b) Clonality D index: iD/uD,
(c) Clonality M index: iM/uM.

The repeatability and linearity tests are detailed in the Supplementary File S1, Table S2a–d and Figure S3a,b.

**Statistical Analysis.** Sample size justification was based on anticipated effect sizes from preliminary data. To detect ≥40% absolute difference in disease progression by comparing patients with normal vs. abnormal FLC M–D patterns (e.g., 15% vs. 55%) with 80% power (and one-sided α of 0.05), a minimum total sample size of 42 patients was required for the validation cohort. Our final validation set (*n* = 46) was therefore sufficiently powered to assess the predictive efficiency of the assay.

All statistical analyses were performed using Python 3.12.4 (pandas 2.2.2, scipy 1.13.1, lifelines 0.29.0, and statsmodels 0.14.2 libraries). Differences in baseline characteristics were assessed using the Mann–Whitney U test for continuous variables and the Chi-square or Fisher’s exact test for categorical variables. Kaplan–Meier curves were used to compare progression-free survival between groups, with log-rank tests assessing significance. Hazard ratios (HRs) were estimated using Cox proportional hazards regression models. Multivariable logistic regression was used to evaluate associations between MDPA, M-protein > 2 g/dL, and FLC ratio > 20 and clinical and biochemical progression. Analyses were performed in the validation cohort. M-protein and FLC ratio were included to allow comparison with the 2/20/20 model. Bone marrow plasma cell percentage was not included due to incomplete availability across the cohort.

Bone marrow plasma cell percentage (BMPC) was determined using either immunohistochemistry (IHC) or flow cytometry (FC), depending on availability; when both were available, the higher value was used to minimize underestimation of plasma cell burden. For the application of the 2/20/20 model, a patient was considered positive if at least one available risk factor was present (BMPC ≥ 20%, M-protein > 2 g/dL, or FLC ratio > 20). A patient was considered negative only if at least two assessed parameters were negative and no positive parameter was identified. For patients with light chain-only disease, the M-protein component was waived, and the determination was based on the FLC ratio and BMPC parameters only. A *p*-value < 0.05 was considered statistically significant.

## 3. Results

**Clinical and biochemical progression.** The cohort included 108 patients, of whom 68 had MGUS and 40 had SMM. The two groups were similar in age (median 68 years for both; *p* = 0.91), but a higher proportion of MGUS patients were male (74% vs. 56%; *p* = 0.001) (Table 1). As expected, SMM patients exhibited features of higher disease burden at baseline, such as significantly higher M-protein, increased immunoparesis, higher bone marrow (BM) PC content and other variables as shown in Table 1. Other variables, such as cytogenetic abnormalities (FISH), β2-microglobulin and the patient’s medical background, did not differ significantly (Table 1). Consistent with these baseline differences, SMM patients experienced higher rates of both clinical and biochemical disease progression over time compared with MGUS patients (Supplementary File S1, Figure S2).

Of the 108 patients, 26 experienced clinical progression, and 46 had biochemical progression over a median follow-up of 5.2 years (range of 0.5–12.9 years). As shown in Table 2, clinical progression occurred in nine MGUS patients and 17 SMM patients (*p* < 0.01). Similarly, biochemical progression was observed in 22 MGUS and 24 SMM patients (*p* = 0.02). Factors consistent with the IMWG 2/20/20 risk model were distributed as follows: M-protein > 2 g/dL and FLC ratio > 20 were significantly associated with both clinical progression (*p* < 0.001 and *p* = 0.002, respectively) and biochemical progression (*p* < 0.0001 and *p* = 0.05). In contrast, BMPC >20% reached statistical significance for clinical progression (15.4% vs. 2.4%, *p* = 0.03) but did not reach significance for biochemical progression (6.5% vs. 5.4%, *p* = 1).

**Figure 1:**
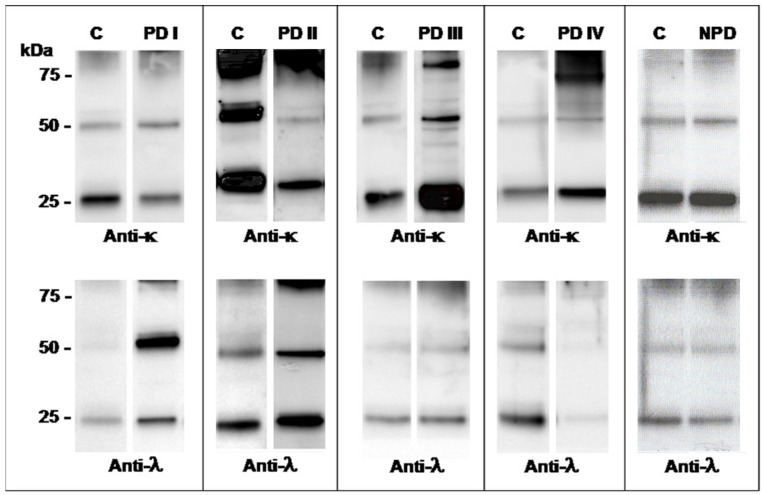
Serum FLC monomer–dimer pattern abnormalities in high-risk MGUS and SMM. Representative SDS-PAGE-based Western blots showing normal FLC monomer (25 kDa) and dimer (50 kDa) patterns in healthy control serum (C), compared with characteristic abnormalities in patients who later progressed (PD). These include increased involved FLC dimerization (PD I, sample #765), disproportionate dimer clonality (PD II, #700), elevated involved monomers (PD III, #758), and specific κ-chain immunoreactivity in the >50–75 kDa region (PD IV, #763). No abnormalities are seen in non-progressors (NPD, #2). The original western blot data of Figure 1 is presented in Supplementary File S2.

**Disease progression by MDPA criteria.** Of the 96 patients’ FLC-MDPA analyzed, 59 showed FLC patterns similar to those observed in a healthy state; the remaining 37 cases showed various FLC M–D pattern abnormalities. Some of these abnormalities were typical of those observed by us previously in AL or active MM [8,9], such as increased dimerization (iD/iM) and high clonality of dimers (iD/uD). The obtained quantification data are presented in the Supplementary File S1. Other pathological changes included highly increased levels of involved monomers or profound specific immunoreactivity in the MW area between >50 and 75 kDa (Figure 1).

Based on MDPA data and biochemical progression outcome in the training set patients (*n* = 50), the MDPA criteria for prediction of PD were established. Specifically, PD can be predicted when at least one of the following criteria is fulfilled:

Criteria I: iD/iM ≥ 3 or iD/uD ≥ 10 (high dimerization of involved FLC or high clonality of dimeric FLC);

Criteria II: [clonality D/clonality M] > 2, in case when iD/iM < 3 and iD/uD from >2 to <10;

Criteria III: iM ≥ 10 (high level of involved monomers);

Criteria IV: iFLC immunoreactivity spreading from >50 to 75 kDa.

The established FLC-MDPA criteria were applied to evaluate additional 46 high-risk samples in the validation set. Comparison of normal vs. abnormal FLC M–D pattern distributions showed similar distributions between the training and validation cohorts for both clinical or/and biochemical progression (Table S3). Likewise, the overall distribution of FLC M–D patterns appeared comparable between clinical and biochemical progression groups, indicating consistent MDPA behavior across outcome definitions.

**Figure 2:**
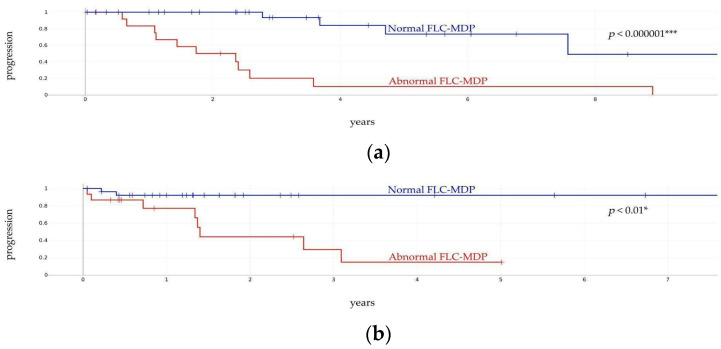
Progression-free survival in patients with normal and abnormal FLC M–D patterns. (**a**): Biochemical progression (normal vs. abnormal FLC M–D patterns, *p* < 0.00001). (**b**): Clinical progression (normal vs. abnormal FLC M–D patterns, *p* < 0.01). * *p* ≤ 0.05; *** *p* ≤ 0.0001.

Abnormal FLC M–D patterns were strongly associated with clinical progression (84% vs. 21%, *p* < 0.0001) and biochemical progression (79% vs. 8%, *p* < 0.000001) (Table 2). FLC-MDPA showed strong predictive performance for biochemical progression (sensitivity 0.79, specificity 0.92, NPV 0.86; HR 19.96, 95% CI 4.31–92) and for clinical progression (sensitivity 0.84, specificity 0.79; HR 13.52, 95% CI 3.68–49.64) (Table 3). Figure 2 shows Kaplan–Meier curves stratified according to FLC-MDPA pattern (abnormal vs. normal), for biochemical (Figure 2a) and clinical progression (Figure 2b). FLC-MDPA demonstrated clear separation between progressors and non-progressors for both endpoints (Figure 2a, *p* < 0.00001; Figure 2b, *p* < 0.01).

**Incorporation FLC-MDPA into the 2/20/20 model.** We next tested whether replacing the bone marrow plasma cell (BMPC ≥ 20%) component of the 2/20/20 model with the FLC-MDPA progression criterion (creating a 2/20/MDPA model) would improve risk stratification (Table 3). In this modified approach (2/20/MDPA), the BMPC criterion was replaced by the MDPA-based progression criterion.

For clinical progression, this substitution modestly increased sensitivity (0.92 vs. 0.88) with essentially unchanged negative predictive value (0.95 vs. 0.94), at the cost of a small reduction in specificity (0.56 vs. 0.61). The impact was more pronounced for biochemical progression, where 2/20/MDPA substantially improved sensitivity (0.93 vs. 0.78) and negative predictive value (0.95 vs. 0.82), while specificity and positive predictive value remained comparable between models (specificity 0.72 vs. 0.70; PPV 0.66 vs. 0.65), without statistically significant differences.

**Figure 3:**
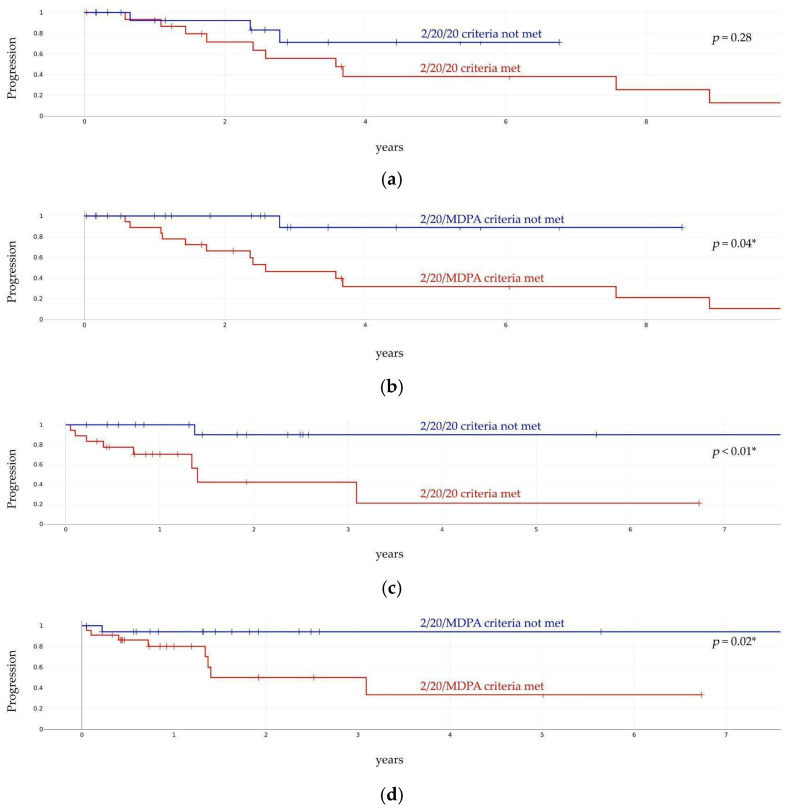
Progression-free survival according to risk assessment: Biochemical: (**a**) 2/20/20 risk assessment and (**b**) the modified 2/20/MDPA model. Clinical: (**c**) 2/20/20 risk assessment and (**d**) the modified 2/20/MDPA model. * *p* ≤ 0.05.

Figure 3 shows Kaplan–Meier curves comparing the classical 2/20/20 model with the modified 2/20/MDPA model. For biochemical progression, the classical model showed no significant separation (Figure 3a, *p* = 0.28), whereas the modified model demonstrated significant separation (Figure 3b, *p* = 0.04). For clinical progression, significant separation was observed for both the classical (Figure 3c, *p* < 0.01) and modified models (Figure 3d, *p* < 0.01), without a clear difference between them.

**Comparison of risk models (MDPA, 2/20/20 and 2/20/MDPA).** In this high-risk enriched cohort, hazard ratios for the three models are summarized in Table 3. For clinical progression, MDPA showed the highest risk discrimination with a hazard ratio of 13.52 (95% CI 3.68–49.64), compared with 5.73 (95% CI 1.23–26) for the 2/20/20 model and 6.8 (95% CI 1.6–28.5) for the 2/20/MDPA model. Significant separation of progression-free survival curves was observed in all three models, without a clear difference between them.

For biochemical progression, MDPA again showed the highest hazard ratio (19.96; 95% CI 4.31–92), compared with 2.07 (95% CI 0.55–7.84) for the 2/20/20 model and 9.89 (95% CI 1.28–76.67) for the 2/20/MDPA model. In contrast to clinical progression, significant separation of survival curves was observed for MDPA (*p* < 0.000001) and for the modified 2/20/MDPA model (*p* = 0.04), whereas no significant separation was observed for the classical 2/20/20 model (*p* = 0.28). These findings are reflected in the Kaplan–Meier plots (Figure 3).

**Multivariable analysis of predictors of progression.** The results of the multivariable multinomial logistic regression are summarized in Table 4. In a three-variable model including MDPA, M-protein > 2 g/dL, and FLC ratio > 20, none of the variables were independently associated with clinical progression, and odds ratios for biochemical progression were not estimable. In a two-variable model excluding M-protein, MDPA was associated with both clinical (*p* = 0.001) and biochemical progression (*p* = 0.04), whereas FLC ratio > 20 was not associated with progression.

## 4. Discussion

In this prospective cohort of MGUS and SMM patients, we evaluated the ability of serum FLC-MDPA as a non-invasive tool in predicting disease progression and possibly replacing the need for a bone marrow examination. Abnormal FLC dimerization patterns were consistently associated with increased risk of both biochemical and clinical progression, supporting the concept that qualitative structural changes in secreted FLCs may reflect a more unstable or advanced precursor clonal state. Patients with abnormal dimerization patterns showed markedly higher progression rates over time, with highly significant separation of progression-free survival, underscoring that MDPA captures clinically meaningful risk stratification beyond random variability (Figure 2). This is consistent with the broader momentum in the field toward minimizing reliance on bone marrow sampling: The Icelandic population-based iStopMM screening study, the largest MGUS screening effort to date (>75,000 individuals), has similarly prioritized the development of peripheral blood-based prediction models to determine which patients require bone marrow biopsy at all [20], underscoring the clinical demand for validated non-invasive tools such as FLC-MDPA.

Several complementary approaches are currently being explored to reduce reliance on bone marrow-based risk assessment. Mass spectrometry-based methods, including quantitative immunopurification-mass spectrometry (QIP-MS) and MALDI-TOF-based MASS-FIX, have substantially improved the sensitivity of monoclonal protein and free light chain detection compared with conventional electrophoretic techniques, and have shown prognostic value for residual disease and progression risk [21,22]. Notably, light chain N-glycosylation identified on routine MASS-FIX testing has recently been reported as an independent structural risk factor for MGUS progression [23], a finding conceptually analogous to our own observation that qualitative, post-translational changes in FLC structure—rather than quantitative FLC burden alone—may signal early malignant transformation. FLC-MDPA and these mass spectrometry-based approaches therefore represent complementary, non-mutually exclusive strategies converging on the same principle: that structural or molecular abnormalities in secreted immunoglobulin components precede and may better predict clinical progression than static quantitative thresholds.

MDPA demonstrated strong predictive performance for both biochemical and clinical progression. For biochemical progression, it achieved high specificity alongside good sensitivity and was associated with a markedly increased risk of progression. For clinical progression, MDPA similarly showed high sensitivity with meaningful specificity, and was associated with a substantially elevated hazard of progression. Importantly, the assay’s consistently high negative predictive value suggests that its greatest clinical utility may be in identifying patients at particularly lower long-term risk, potentially enabling less intensive follow-up and reducing unnecessary invasive evaluations in selected patients.

We further examined whether MDPA could enhance established clinical risk models by integrating it into a modified 2/20/20 framework, substituting the bone marrow plasma cell burden component with MDPA-defined progression criteria. While the original Mayo criteria were tested for only clinical progression within two years [19], we extrapolated this to a longer follow-up and for biochemical progression as well. This modification improved sensitivity and maintained a high negative predictive value for both clinical and biochemical progression, while preserving overall performance characteristics comparable to the original model. Notably, the modified model provided clearer separation of biochemical progression-free survival compared with the original 2/20/20 model, suggesting that MDPA captures risk information that is not fully represented by bone marrow–based measurements alone.

Our approach of enriching the 2/20/20 model parallels a separate, recent direction in the field: Rather than substituting individual static parameters, the recently reported PANGEA-SMM model demonstrated that incorporating the trajectory of standard biomarkers over time—including the rate of change in M-protein, FLC ratio, hemoglobin, and creatinine—outperformed both the 2/20/20 and IMWG genomic models in a large multinational cohort [24]. This suggests that risk information not captured by cross-sectional measurements may be recoverable either through longitudinal tracking of conventional markers, as in PANGEA-SMM, or through a single structural assay such as FLC-MDPA that may already reflect an evolving clonal state. Serial FLC-MDPA testing, analogous to the dynamic biomarker approach, represents a natural extension of the present work and should be evaluated in future longitudinal cohorts.

Thus, MDPA may replace the need for invasive workup. Clinically, FLC-MDPA may serve several complementary roles beyond a single biopsy decision. First, at the point where a bone marrow biopsy is being considered—particularly in patients with intermediate- or high-risk features by conventional criteria—a negative MDPA result, given its high sensitivity and negative predictive value, could support deferring bone marrow evaluation in favor of close biochemical monitoring, whereas a positive result would reinforce the indication for biopsy and IMWG-based workup. Second, independent of the biopsy decision, MDPA status may help guide follow-up intensity: patients with a negative result and low overall risk could reasonably be monitored at standard intervals, while a positive result—reflecting a marked increase in progression risk in this cohort—may warrant closer biochemical and clinical surveillance even in the absence of an immediate indication to treat. Third, when integrated into the modified 2/20/MDPA model, MDPA-based risk stratification could inform treatment timing: a high-sensitivity, high-NPV negative result may support continued observation, whereas a positive result, taken together with established IMWG treatment criteria, may support earlier consideration of therapy or enrollment in early-intervention clinical trials. In this way, MDPA may function less as a stand-alone replacement for bone marrow assessment and more as an adjunct that helps triage biopsy need, calibrate follow-up frequency, and support treatment-timing decisions alongside existing risk models.

This study has limitations. Our cohort was enrolled using established high-risk criteria that overlap with several 2/20/20 components, which restricts the range of these parameters in our dataset and may attenuate the model’s apparent performance relative to an unselected population. As a result, the hazard ratio comparisons presented here should be interpreted as reflecting FLC-MDPA’s performance within an already-identified high-risk cohort, rather than a definitive head-to-head comparison in an unselected population. Additionally, the cohort size was modest, and the two-stage design (training followed by validation) resulted in shorter follow-up for the validation cohort, which likely reduced statistical power and limited the precision of effect estimates. This is well reflected in the multivariate analysis. In addition, although MDPA relies on standard Western blotting methodology, it has thus far been implemented in a single laboratory; broader clinical translation will require inter-laboratory standardization, reproducibility testing, and protocol harmonization to ensure scalability and consistent interpretation. Finally, while sensitivity and negative predictive value were high, specificity and positive predictive value were more modest, indicating that MDPA may be best positioned as a rule-out biomarker rather than a stand-alone rule-in test.

## 5. Conclusions

In summary, FLC-MDPA is a promising laboratory tool helping prediction of disease progression in MGUS and SMM and improving sensitivity and negative predictive values when incorporated into a modified clinical risk framework. If validated in larger independent cohorts, MDPA could support a biomarker-driven monitoring strategy that better identifies low-risk patients, reduces reliance on bone marrow evaluation, and enables more individualized follow-up intensity. Future studies should confirm these findings, refine assay thresholds, establish cross-laboratory reproducibility, and determine whether MDPA can guide monitoring intervals or inform early intervention strategies in precursor plasma cell disorders.

## Figures and Tables

**Table 1:** Patient baseline characteristics by plasma cell disorder.

Variable		MGUS (*N* = 68)	SMM (*N* = 40)	*p*
Age (median)		68 (42–88)	68 (34–92)	0.91
Gender	Male (*n*, %)	50 (74.6%)	23 (56.1%)	0.001 **
	Female (*n*, %)	18 (43.9%)	17 (25.4%)	
M-Protein Type	IgA (*n*, %)	17 (25%)	13 (33%)	<0.001 **
	IgG (*n*, %)	18 (27%)	22 (55%)	
	IgM (*n*, %)	9 (13%)	0 (0%)	
	LC (*n*, %)	24 (34%)	5 (12%)	
LC Type	κ (*n*, %)	43 (63%)	26 (65%)	0.84
	λ (*n*, %)	25 (37%)	14 (35%)	
Immunoglobulin (mg/dL)	IgA (Median)	796 (482–3516)	1290 (482–3952)	0.01 *
	IgG (Median)	1870 (1500–2974)	2594 (1513–7470)	0.02 *
	IgM (Median)	1600 (155–1734)	-	-
FLC (mg/L)	FLC κ (Median)	72.9 (6.61–394)	58 (10.72–994)	0.55
	FLC λ (Median)	30.0 (2.29–617)	15.2 (1.59–814)	0.08
FLC Ratio (Median)		4.9 (1.02–670.0)	6.3 (1.05–72.2)	0.41
dFLC (Median)		88.9 (2.6–571.0)	91.7 (3.2–962.6)	0.48
M-Protein (Median, g/dL)		0.52 (0.03–3.52)	1.37 (0.03–7.47)	0.001 **
Immunoparesis (*n*, %)		19 (28%)	22 (56%)	<0.01 *
Albumin (Median, g/dL)		4.2 (3.5–5.2)	4.1 (3.5–4.6)	0.32
Creatinine (Median, mg/dL)		1.06 (0.01–2.88)	0.89 (0.61–2.43)	0.04 *
β2 microglobulin (Median, mg/L)		3.37 (0.4–12.1)	4 (0.7–10.0)	0.42
BMPC% (Median, IHC)		7.5 (3–20)	15 (3–70)	<0.01 *
BMPC% (Median, FC)		1.05 (0.12–9)	4.7 (0.3–36)	0.02 *
FISH CA HR (*n*, %)		4 (6%)	4 (10%)	0.95
Aberrant cell > 95% (*n*, %)		8 (11.7%)	7 (18%)	0.96

*Note: p*-values calculated from Mann–Whitney U test for continuous variables or Fisher’s exact test for categorical variables. Abbreviations: MGUS, monoclonal gammopathy of undetermined significance; SMM, smoldering multiple myeloma; LC, light chain; FLC, free light chain; dFLC, difference between involved and uninvolved free light chains; BMPC, bone marrow plasma cell percentage; IHC, immunohistochemistry; FC, flow cytometry; FISH CA HR, high-risk cytogenetics by fluorescence in situ hybridization. * *p* ≤ 0.05; ** *p* ≤ 0.001.

**Table 2:** Baseline characteristics by progression status (clinical and biochemical).

Variable		Clinical Progression (Yes/No)		*p*	Biochemical Progression (Yes/No)		*p*	*p* ^a^
		No PD (*N* = 82)	PD (*N* = 26)		No PD (*N* = 56)	PD (*N* = 46)		
Gender	Male (*n*, %)	56 (68%)	12 (46%)	0.06	38 (68%)	25 (54%)	0.18	0.8
	Female (*n*, %)	26 (32%)	14 (54%)		18 (32%)	21 (46%)		
PCD	MGUS (*n*, %)	59 (72%)	9 (35%)	<0.01 *	40 (71%)	22 (48%)	0.02 *	0.8
	SMM (*n*, %)	23 (28%)	17 (65%)		16 (29%)	24 (52%)		
Age, years (Median)		69 (42–92)	67 (34–86)	0.91	67 (42–92)	71 (34–91)	0.02 *	0.68
M-Protein Type	IgA (*n*, %)	21 (26%)	9 (35%)	0.66	22 (39%)	7 (15%)	0.04 *	0.99
	IgG (*n*, %)	30 (37%)	10 (38%)		17 (30%)	23 (50%)		
	IgM (*n*, %)	8 (10%)	1 (4%)		5 (9%)	3 (7%)		
	LC (*n*, %)	23 (28%)	6 (23%)		12 (21%)	13 (28%)		
LC Type	κ (*n*, %)	56 (68%)	23 (88%)	0.03 *	42 (75%)	33 (72%)	0.88	0.59
	λ (*n*, %)	26 (32%)	3 (12%)		14 (25%)	13 (28%)		
M-Protein (Median, g/dL)		1.02 (0.03–4.98)	3.30 (0.03–7.47)	<0.001 **	0.9 (0.03–4.98)	2.2 (0.03–7.47)	<0.001 **	0.94
Immunoglobulin (mg/dL)	IgA (Median)	800 (482–1807)	1830 (482–3952)	<0.001 **	911 (482–3952)	1715 (482–3205)	0.15	0.87
	IgG (Median)	2147 (1500–4978)	3049 (1536–7470)		1787 (1513–2800)	2730 (1500–7470)		
	IgM (Median)	1635 (155–1734)	1600 (1600–1600)		1525 (155–1670)	1734 (1600–1734)		
FLC (mg/L)	FLC κ (Median)	48.7 (6.61–592)	83.2 (13.10–1246)	0.04 *	46.27 (6.61–592)	94 (7.39–1246)	0.07	0.99
	FLC λ (Median)	24.29 (0.83–814)	14.1 (1.59–617)		21.06 (0.83–418)	21.82 (1.59–814)		
FLC Ratio (Median)		5 (1–80)	8 (1–89)	0.05 *	3 (1–80)	11.33 (1–89)	<0.001 **	0.79
dFLC (Median)		88.9 (2.6–571.0)	91.7 (3.2–962.6)	0.48	77.3 (2.6–571.0)	165.6 (11.5–962.6)	0.06	0.99
Creatinine (Median, mg/dL)		1 (0.53–2.88)	0.91 (0.64–4.18)	0.41	0.99 (0.53–4.18)	0.97 (0.61–2.024)	0.84	0.32
Albumin (Median, g/dL)		4.2 (3.5–5.2)	4 (3.5–4.8)	0.02 *	4.2 (3.5–5.2)	4.1 (3.5–4.8)	0.31	0.98
β2 microglobulin (Median, mg/L)		4.03 (0.01–7217.0)	3.43 (0.00–12,093)	0.72	3.75 (0.01–12,093.0)	3.43 (0.00–10,099)	0.41	0.71
M-Protein > 2 (*n*, %)		14 (17.5%)	15 (57%)	<0.001 **	7 (12.3%)	22 (47%)	<0.0001 ***	0.88
FLC Ratio > 20 (*n*, %)		7 (8.8%)	9 (34%)	0.01 *	5 (8.8%)	11 (23%)	0.05 *	1
BMPC > 20% (*n*, %)		2 (2%)	4 (15%)	0.03 *	3 (5%)	3 (6%)	1	1
BMPC % (Median, IHC)		5 (3–50%)	15 (5–70.0%)	<0.01 *	7.5 (3–70%)	8 (3–50%)	0.45	0.15
BMPC % (Median, FC)		1 (0.12–6%)	7.5 (0.28–36%)	<0.001 **	1.7 (0.12–36%)	2 (0.2–20%)	0.34	0.09
FISH CA HR (*n*, %)		4 (5%)	5 (19%)	0.04 *	0 (0%)	5 (11%)	0.02 *	0.41
Immunoparesis (*n*, %)		29 (34%)	12 (46%)	0.36	24 (42.1%)	17 (37%)	0.69	0.78
Aberrant cell > 95% (*n*, %)		9 (10%)	4 (15%)	0.51	6 (10%)	8 (17%)	0.39	0.84
Progression to MM (*n*, %)		2 (2.5%)	21 (80%)	<0.0001 ***	4 (7.0%)	19 (41%)	<0.001 **	0.87
Normal FLC M–D pattern ^b^ (*n*, %)		26 (79%)	2 (15%)	<0.0001 ***	24 (92%)	4 (21%)	<0.000001 ***	1
Abnormal FLC M–D pattern ^b^ (*n*, %)		7 (21%)	11 (84%)		2 (8%)	15 (79%)		

*Note*: *p*-values calculated from Mann–Whitney U test for continuous variables or Fisher’s exact test for categorical variables. Abbreviations: PD, progression disease; PCD, plasma cell disorder; MGUS, monoclonal gammopathy of undetermined significance; SMM, smoldering multiple myeloma; FLC, free light chain; dFLC, difference between involved and uninvolved free light chains; BMPC, bone marrow plasma cell percentage; IHC, immunohistochemistry; FC, flow cytometry; FISH CA HR, high-risk cytogenetics by fluorescence in situ hybridization. ^a^ *p* clinical status vs. biochemical status. ^b^ FLC-MDPA data of the validation set patients (*n* = 46). * *p* ≤ 0.05; ** *p* ≤ 0.001; *** *p* ≤ 0.0001.

**Table 3:** Prediction of PD using MDPA, 2/20/20 and 2/20/MDPA models.

Metrics	Prediction of Clinical Progression			Prediction of Biochemical Progression		
	MDPA	2/20/20	2/20/MDPA	MDPA	2/20/20	2/20/MDPA
Sensitivity	0.84	0.88	0.92	0.79	0.78	0.93
Specificity	0.79	0.61	0.56	0.92	0.7	0.72
PPV	0.61	0.44	0.44	0.88	0.65	0.66
NPV	0.93	0.94	0.95	0.86	0.82	0.95
Log-rank *p* value	<0.01 *	<0.01 *	0.02 *	<0.000001 ***	0.28	0.04 *
HR (C.I.)	13.52 (3.68–49.64)	5.73 (1.23–26)	6.8 (1.6–28.5)	19.96 (4.31–92)	2.07 (0.55–7.84)	9.89 (1.28–76.67)

*Note*: Log-rank *p*-values were derived from Kaplan–Meier analysis; hazard ratios from Cox regression. Abbreviations: MDPA, monomer–dimer patterns analysis; PPV, positive predictive value; NPV, negative predictive value; HR (CI), hazard ratio (confidence interval). * *p* ≤ 0.05; *** *p* ≤ 0.0001.

**Table 4:** Multinomial logistic regression analysis of predictors of clinical- and biochemical-based progression.

	Variable	Clinical Progression OR (95% CI)	*p*	Biochemical Progression OR (95% CI)	*p*
3-Variable Model	M-protein > 2 g/dL	5.79 (0.45–73.71)	0.18	Not estimable	—
	FLC ratio > 20	4.03 (0.44–36.96)	0.22	Not estimable	—
	MDPA	4.03 (0.44–36.96)	0.22	Not estimable	—
2-Variable Model	FLC ratio > 20	4.94 (0.61–39.87)	0.13	1.02 (0.06–17.9)	0.99
	MDPA	8.21 (1.12–60.25)	0.04 *	74.69 (5.65–987.61)	0.001 **

*Note*: Multinomial logistic regression assessing independent predictors of clinical- and biochemical-based progression. OR—odds ratio, CI—confidence interval. * *p* ≤ 0.05; ** *p* ≤ 0.001.

## Data Availability

The raw data supporting the conclusions of this article will be made available by the authors on request.

## Supplementary Materials

The following supporting information can be downloaded at https://www.mdpi.com/article/10.3390/cancers18152409/s1. Supplementary File S1: Figure S1. Study flow chart. Of 108 enrolled patients, 96 with complete clinical data underwent FLC-MDPA and follow-up. Fifty patients (training set) were used to refine the assay, and 46 (validation set) were analyzed to test its predictive performance. Figure S2. Progression free survival of all patient cohort: SMM (*n* = 40) MGUS (*n* = 68) patients. (a) biochemical progression (*p* = 0.01) and (b) clinical progression (*p* < 0.01) for SMM compared with MGUS patients. Table S1a. Quantitative M-D pattern analysis in sera of NDP patients. Table S1b. Quantitative M-D pattern analysis in sera of PD patients. Table S2a. SD_r_ values estimated for normalized and monomers and dimers D) values (FLC indices). Table S2b. SD_IP_ estimated using normalized and monomers and dimers D) values. Table S2c. SD_IP diagnostic_ estimated for the diagnostic FLC index values: Clonality D, Clonality M, Clonality D/Clomality M, as well as κ and λ dimerization indices. Figure S3a. Primary intensity signals (not normalized) of κ and λ FLC dimeric (D) and monomeric (M) bands at the different serum sample dilutions with PBS. Table S2d. Primary intensity signals at different serum dilutions (relative FLC concentrations). Figure S3b. FLC WB analysis of serum samples of two patients with monoclonal gammopathy (#83 and #84) and control sample (C). Abnormally high intensity of involved FLC monomers and dimers (κ in sample #83 and λ in sample #84) and strong supression of uninvoved FLC (λ in sample #83 and κ in sample #84) are demonstrated. Table S3. Patient Medical Background by Plasma Cell Disorder. Table S4. Comparison of FLC Monomer–Dimer Pattern Distributions Between Training and Validation Cohorts According to Clinical and Biochemical Disease Progression. Refs. [25,26,27,28,29] are cited in the Supplementary Materials. Supplementary File S2: Original images for blots presented in the manuscript, Figure 1.

## Abbreviations

The following abbreviations are used in this manuscript:

AL	Amyloid light chain amyloidosis
BM	Bone marrow
BMPC	Bone marrow plasma cells
CRAB	Hypercalcemia, renal insufficiency, anemia, bone lesions
dFLC	Difference between involved and uninvolved free light chains
FLC	Free light chain
FLC-MDPA	Free light chain-monomer–dimer pattern analysis
FC	Flow cytometry
HR	Hazard ratio
IHC	Immunohistochemistry
IMWG	International Myeloma Working Group
MGUS	Monoclonal gammopathy of undetermined significance
MM	Multiple myeloma
MRI	Magnetic resonance imaging
NPD	Non-progressive disease
PC	Plasma cells
PCD	Plasma cell disorders
PD	Progressive disease
SMM	Smoldering multiple myeloma
WB	Western blot
