# Supplementary material for: Free Light Chain Monomer—Dimer Pattern Analysis as Non-Invasive Tool in Predicting MGUS and SMM Progression
Source: Cancers (Basel). 2026 Jul 26;18(15):2409. doi: 10.3390/cancers18152409 (PMC13464981; doi:10.3390/cancers18152409)
Supplement: Supplementary file 1 [file cancers-18-02409-s001.zip › cancers-4380877-supplementary.pdf]

Supplementary File S1

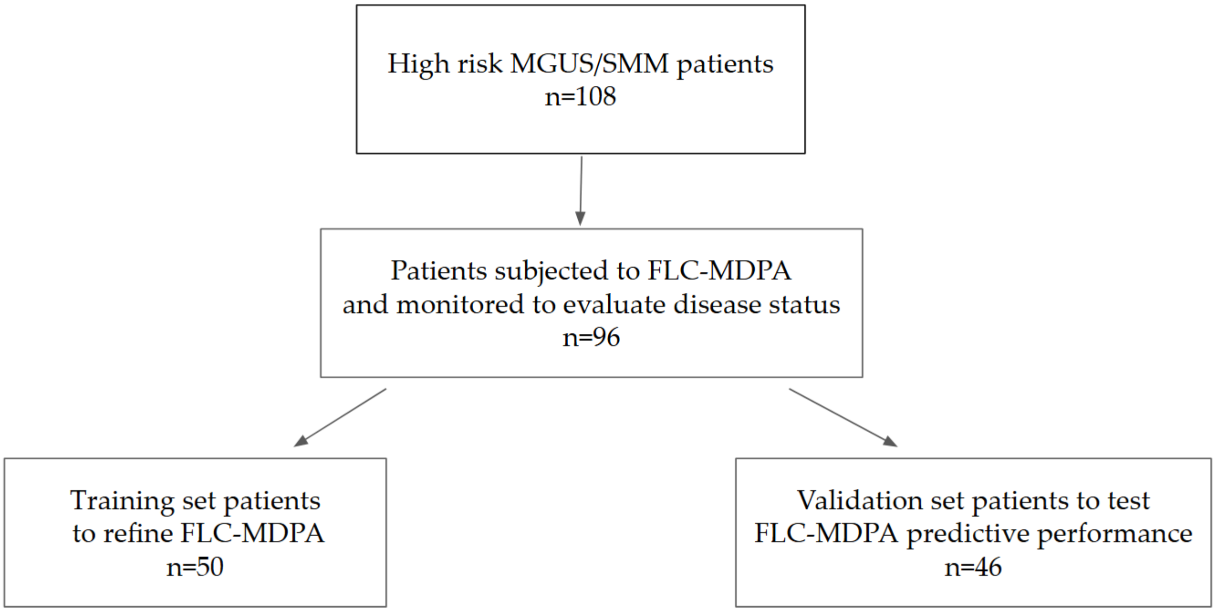

Figure S1. Study flow chart. Of 108 enrolled patients, 96 with complete clinical data underwent FLC-MDPA and follow-up. Fifty patients (training set) were used to refine the assay, and 46 (validation set) were analyzed to test its predictive performance.

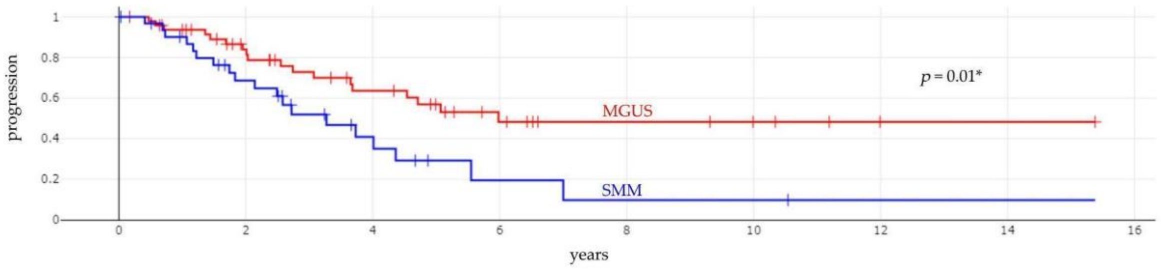

(a)

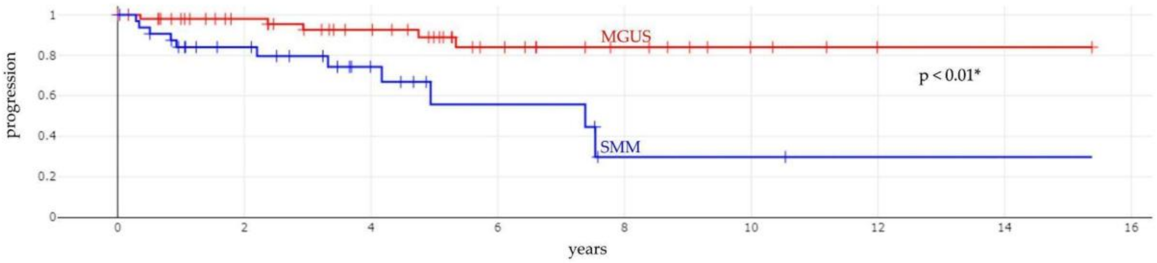

(b)

Figure S2. Progression free survival of all patient cohort: SMM (n=40) MGUS (n=68) patients. (a) biochemical progression (p = 0.01) and (b) clinical progression (p < 0.01) for SMM compared with MGUS patients

Supplement Table S1a. Quantitative M-D pattern analysis in sera of NDP patients

Sample code	M-D pattern	λD/λM	κD/κM	κD/λD	λD/κD	κD/ λD divided by κM/λM	λD/κM divided by λM/κM	κM	**λM**
2	Normal	1.1	0.9	0.9	1.11	0.8	1.25	1.23	1.1
10	Normal	1.2	0.83	0.22	4.55	0.7	1.43	0.89	2.8
16	Abnormal	3.8	1.12	0.1	10.0	0.33	3.03	0.60	1.8
22	Abnormal	1.16	0.32	0.27	3.6	0.27	3.6	1.3	1.3
26	Normal	0.6	0.37	8.0	0.13	0.59	1.70	4.29	0.32
39	Normal	1.57	0.93	0.41	2.44	0.59	1.69	1.24	1.8
702	Normal	0.4	1.67	1.65	0.61	0.4	2.50	2.2	0.52
710	Normal	0.61	0.39	3.55	0.28	0.39	2.56	2.85	0.51
721	Abnormal	Very high (immeasurable) lambda dimer							
746	Normal	0.5	0.42	0.38	2.6	1.19	0.52	0.32	0.35
752	Normal	0.74	0.14	3.7	0.27	0.19	5.26	2.6	0.68
768	Normal	1.5	0.68	3.9	0.26	0.4	2.50	3.57	0.41
774	Normal	1.19	0.67	2.9	0.34	0.56	1.77	0.92	0.18
785	Normal	1.37	0.69	0.9	1.11	0.53	1.89	1.45	0.82
787	Normal	2.32	1.0	1.64	0.61	0.43	2.33	2.5	0.66
791	Normal	1.9	0.58	1.82	0.55	0.30	3.32	5	0.42
794	Normal	1.53	0.35	1.8	0.56	0.23	4.27	0.93	0.12
800	Normal	2.9	0.54	2.38	0.42	0.185	0.7	2.5	0.194
801	Normal	0.18	0.55	1.36	0.74	0.30	3.30	2.5	0.55
805	Normal	0.9	0.59	1.29	0.78	0.2	5.00	0.6	3.0
810	Normal	0.76	0.5	1.7	0.59	0.66	1.52	2.9	0.87
812	Normal	0.57	0.28	1.78	0.56	0.49	2.04	2.3	0.63

823	Normal	1.39	0.64	1.0	1.00	0.46	2.17	1.1	0.49
830	Normal	0.97	0.63	0.26	3.88	0.64	1.55	1.23	3.0
835	Normal	1.72	1.42	0.26	3.85	0.8	1.25	1.69	5.0
840	Normal	2.6	0.52	0.28	3.62	0.20	4.90	1.43	1.1
733	Normal	1.44	1.1	1.6	0.62	0.76	1.3	1.1	0.6

Note: no specific immunoreactivity was observed in the molecular weight range from >50 kDa to 75 kDa.

Supplement Table S1b. Quantitative M-D pattern analysis in sera of PD patients

Sample code	iFLC	M-D pattern	iD/iM	iD/uD	iD/uD divided by iM/uM	iM	Specific IR >50 – 75 kDa
G86	κ	PD I	0.5	high*	trace**	0.7	-
G106	κ	PD I	0.08	high*	trace**	11.6	-
717	κ	PD II	1.57	2.4	3.1	1.31	-
720	κ	PD I	1.6	20	1.3	2.56	-
743	κ	PD I	0.43	high*	trace**	5.0	-
758	κ	PD III	0.53	1.24	2.35	>10	-
762	κ	PD I	0.54	high*	trace**	2.1	-
763	κ	PD IV	1.14	1.87	1.12	1.13	+
.783	κ	PD I	0.6	12.7	4.2	2.6	-
786	κ	Normal	1.0	4.1	0.66	2.3	-
817	κ	PD I	1.6	>20	3.27	1.0	-
824	κ	Normal	0.4	3.2	0.7	1.0	-
832	κ	PD I	0.89	10	2.1	1.36	-
833	κ	PD IV	0.63	1.5	0.6	1.3	+
700	λ	PD II	1.0	9	2.1	3	-
G84	λ	PD III***					-
765	λ	PD I	8.8	2.17	4.4	1.36	-
767	λ	PD I	3.1	6.2	6.2	1.0	-
797	λ	?	1.6	1.16	12.1	1.68	-
808	λ	PD IV	1.7	7.8	2.0	7.1	+
842	λ	PD III	0.41	0.15	2.6	10.7	-

Abbreviations: FLC – free light chains; i – involved; u – uninvolved; M – monomer. D – dimer;
IR – immunoreactivity; PD – progressive disease.
*high κD/λD ratio value due to the immeasurably weak bands
**immeasurable
***qualitative evaluation: highly increased intensity of λ FLC (especially λ monomer) with a very low intensities (immeasurable) of κ dimeric and monomeric bands (Supplement 1).

Supplement S1
Repeatability

1. Sera from 8 patients were tested. Each sample was tested twice on the same day (on the same gel) by the same operator using the same laboratory equipment. The standard deviation values under repeatability conditions, $\mathbf{SD_r}$, were estimated using normalized and monomers and dimers D) values (FLC indices).Two approaches were used to evaluate the $\mathbf{SD_r}$. The first approach is based on the estimation of the average range between duplicate measurements. This approach is widely used in quality control for the small samples (m ≤ 10) [1]. The following formula for $\mathbf{SD_r}$ calculation was used:

$$SD_r = \frac{R_{Av}}{d_{m,n}} SD_r = \frac{R_{Av}}{d_{m,n}},$$

where,

$\boldsymbol{R_{av}}$ – the average range between duplicate measurements,

$\boldsymbol{m}$ - number of the measured pairs (m = 7 or 8),

$\boldsymbol{n}$ – number of repeated measurements (n = 2),

$\boldsymbol{d_{m,n}}$ – coefficient taking into consideration m and n.

In our case, $\boldsymbol{d_{7,2}}$ = 1.17 and $\boldsymbol{d_{8,2}}$ = 1.16 [2].

The second approach uses the Dahlberg formula [3,4] for the estimation of the $\mathbf{SD_r}$

$$\boldsymbol{SD_r = \sqrt{\sum\frac{(x' - x'')^2}{2m}} SD_r = \sqrt{\sum\frac{(x' - x'')^2}{2m}}}$$

where,

$\boldsymbol{m}$ –number of paired measurements (under repeatability conditions),

$\boldsymbol{x'}$ and $\boldsymbol{x''}$ – results of the paired measurements.

Supplement Table S2a. $\mathbf{SD_r}$ values estimated for normalized and monomers and dimers D) values (FLC indices).

		κD	κD	κM	λD
Estimation of SD_r via average range	SD_r	0.134	0.049	0.124	0.053
Dahlberg method	SD_r	0.158	0.051	0.132	0.056

2. Intermediate precision (IP) was evaluated by analyzing FLC in serum samples of 10 patients. Each sample was tested twice on *different days* and *different gels* by the same operator using the same laboratory equipment. In other words, the data were obtained under the intermediate precision conditions. The two same statistical approaches used to estimate $\mathbf{SD_r}$, were also used to evaluate the intermediate precision, $\mathbf{SD_{IP}}$. As expected, the $\mathbf{SD_{IP}}$ values for all the four FLC indices, estimated under the intermediate precision conditions are higher than the corresponding $\mathbf{SD_r}$ values obtained under repeatability conditions.

(A) The intermediate precision (**IP)** standard deviation values, **$SD_{IP,}$** were estimated for each of four FLC indices, **κD**, **κM**, **λD**, **λM** : **SD_r,** were estimated using normalized and monomers and dimers D) values (FLC indices) (Table S3b).

Supplement Table S2b. SD_{IP} estimated using normalized and monomers and dimers D) values

		κD	κD	κM	λD
Estimation of SD via average range	SD_{IP}	0.219	0.221	0.260	0.283
Dahlberg method	SD_{IP}	0.205	0.199	0.366	0.289

(B) In case of the *diagnostic* FLC indices, the intermediate precision standard deviation values (**$SD_{IP\ diagnostic}$**) were estimated for the index values of Clonality D, Clonality D/ Clomality M, as well as for κ and **λ** dimerization indices (Table S3c).

Supplement Table S2c. $SD_{IP\ diagnostic}$ estimated for the diagnostic FLC index values: Clonality D, Clonality M, Clonality D/ Clomality M, as well as κ and **λ** dimerization indices.

		$κD_N/κM_N$	$λD_N/λM_N$	$κD_N/λD_N$	Clonality D / Clonality M
Estimation of SD via average range	$SD_{IPdiagnostic}$	0.238	0.308	0.142	0.111
Dahlberg method	$SD_{IPdiagnostic}$	0.262	0.309	0.138	0.114

<u>Note.</u> The values of **$SD_{IP\ diagnostic}$** obtained using two different approaches are remarkably similar.

Finally, the estimated values of $SD_{IP\ diagnostic}$ meet the commonly accepted criteria for bioassay [5].

2. Linearity

The concentrations of the serum FLC vary significantly among the patients with monoclonal gammopathy. Therefore, there was a need to establish the linear range relationship between the FLC concentrations (serum dilutions) and the corresponding intensity signals generated by the immunoreactive bands. We have measured the signals at different dilutions of our reference serum sample comprising a mixture of sera samples of 15 healthy individuals. Assumption was made that the relative FLC concentrations are directly proportional to the degree of dilution.

The below displayed Fig. S3a and Table S2d demonstrate intensity signals (primary signals) generated by κ and λ FLC dimeric (D) and monomeric (M) bands at the different serum sample dilutions with PBS expressed as percentage of serum concentration in PBS: track 1 - 4.38%, track 2 – 3.75 %, track 3 - 3.125%, track 4 – 2.5%, track 5 – 1.875%, and track 6 - 1%.

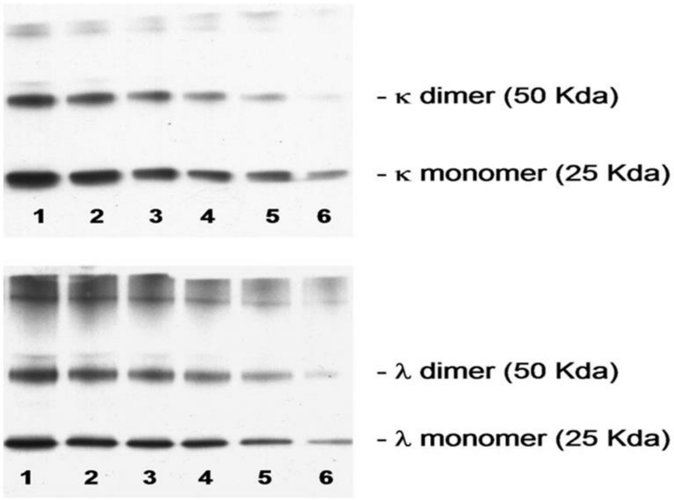

Supplement Figure S3a. Primary intensity signals (not normalized) of κ and λ FLC dimeric (D) and monomeric (M) bands at the different serum sample dilutions with PBS

Supplement Table S2d. Primary intensity signals at different serum dilutions (relative FLC concentrations).

Serum % in PBS, vol/vol	Primary intensity signal of FLC κ and λ monomers (M) and dimers (D)			
	κD	κM	λD	λM
4.38	0.071	0.108	0.077	0.06
3.75	0.053	0.082	0.055	0.048
3.125	0.044	0.06	0.047	0.044
2.5	0.028	0.05	0.03	0.037
1.875	0.015	0.042	0.018	0.021
1	0.004	0.02	0.006	0.011

The dilution range was found to be linear for serum dilution with PBS varying from 1:99 to 1:21 (1% to 4.5% of serum concentration in PBS), thus corresponding to the primary FLC signal intensity (not normalized) varying from 0.005 to 0.07.

In some cases, intensity of involved FLC (i.e, either κ or λ monomers and dimers) is very high, while the intensity of uninvolved FLC is barely measurable (**Supplement Figure S3b**). In such cases the FLC pattern

pathology is obvious and does not require further processing and quantification.

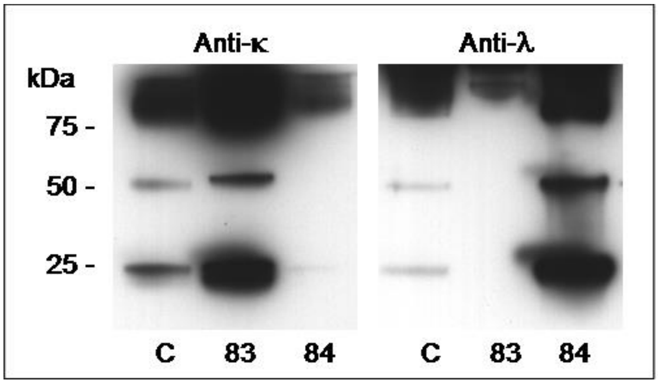

Supplement Figure S3b. FLC WB analysis of serum samples of two patients with monoclonal gammopathy (#83 and #84) and control sample (C). Abnormally high intensity of involved FLC monomers and dimers (κ in sample #83 and λ in sample #84) and strong supression of uninvoved FLC (λ in sample #83 and κ in sample #84) are demonstrated.

1. Massart DL, et al. *Handbook of Chemometrics and Qualimetrics: Part A*; 1997, Elsevier, p.31.
2. David H.A.. Further applications of range to analysis of variance. *Biometrika* (1951) **38**, 393.
3. Dahlberg G. In *Statistical methods for medical and biological students*. London: G. Allen & Unwin Ltd. (1940).
4. Hae-Young K. Statistical notes for clinical researchers: Evaluation of measurement error 2: Dahlberg's error, Bland-Altman method, and Kappa coefficient. *Restor Dent Endod.* 2013; **38**(3): 82–185.
5. Little T.A. Establishing acceptance criteria for analytical methods. *BioPharm International* (2016) **29(10),** 44-48.

All blots, and further guidance will be made available at request with Dr. Batia Kaplan batiakaplan@gmail.com

Table S3 Patient Medical Background by Plasma Cell Disorder

Variable	Value	MGUS (*N=68*)	SMM (*N=40*)	*p*
Metabolic	*(n, %)*	41 (62%)	17 (42%)	
Cardiovascular	*(n, %)*	23 (34%)	15 (37%)	0.7
Pulmonary	*(n, %)*	16 (24%)	9 (22%)	

Variable	Value	MGUS (*N=68*)	SMM (*N=40*)	*p*
Neurological	*(n, %)*	15 (22%)	9 (22%)	
Rheumatological	*(n, %)*	13 (19%)	9 (22%)	
Endocrinological	*(n, %)*	14 (21%)	7 (17%)	
Nephrological	*(n, %)*	15 (22%)	4 (10%)	
Gastrointestinal	*(n, %)*	11 (16%)	7 (17%)	
Urological	*(n, %)*	12 (18%)	6 (15%)	
Hematological	*(n, %)*	13 (19%)	4 (10%)	
Surgical	*(n, %)*	11 (16%)	4 (10%)	
Orthopedic	*(n, %)*	6 (9%)	3 (7%)	
Oncological	*(n, %)*	4 (6%)	3 (7%)	
Immunological	*(n, %)*	3 (4.5%)	3 (7%)	
Infectological	*(n, %)*	3 (4.5%)	3 (7%)	
Dermatological	*(n, %)*	5 (7.6%)	0 (0%)	
Ophthalmological	*(n, %)*	0 (0%)	2 (5%)	
Psychiatric	*(n, %)*	1 (1.5%)	1 (2%)	
Gynecological	*(n, %)*	0 (0%)	1 (2%)	
ENT	*(n, %)*	1 (1.5%)	0 (0%)	

Abbreviations: MGUS, monoclonal gammopathy of undetermined significance; SMM, smouldering multiple myeloma; ENT, ear, nose and throat.

Table S4 Comparison of FLC Monomer–Dimer Pattern Distributions Between Training and Validation Cohorts According to Clinical and Biochemical Disease Progression

Variable	Value	Clinical progression (Yes/No)	*p*	*p''*	Biochemical progression (Yes/No)	*p*	*p''*	*p'*	*p'''*

			No PD (N=37)	PD (N=13)			No PD (N=26)	PD (N=24)				
Training cohort	Normal FLC M-D pattern	*(n, %)*	25(67%)	3 (23%)	< 0.01*		20 (77%)	9 (37%)	< 0.01*		0.46	
	Abnormal FLC M-D pattern	*(n, %)*	9 (24%)	10 (77%)			6 (23%)	15 (63%)				
			**No PD (N=33)**	**PD (N=13)**		0.82	**No PD (N=26)**	**PD (N=19)**		0.68		0.49
Validation cohort	Normal FLC M-D pattern	*(n, %)*	26 (79%)	2 (15%)	< 0.0001***		24 (92%)	4 (21%)	< 0.000001***		0.49	
	Abnormal FLC M-D pattern	*(n, %)*	7 (21%)	11 (84%)			2 (8%)	15 (79%)				

Note: p-values were calculated using Fisher's exact test for categorical variables. p compares the distribution of FLC monomer–dimer (M–D) patterns between patients with and without progression within each cohort. p′ compares the distribution of FLC M–D patterns between clinical and biochemical progression groups. p″ compares the distribution of progression (clinical or biochemical) between the training and validation cohorts. p‴ compares the overall distribution of FLC M–D patterns between the training and validation cohorts.
Abbreviations: PD, progressive disease; FLC, free light chain; M–D, monomer–dimer; MDPA, monomer–dimer pattern analysis. * $p \leq 0.05$; ** $p \leq 0.001$; *** $p \leq 0.0001$.

Supplementary File S2

Original images for blots presented in the manuscript, Fig.1

` Anti-κ: PD 1 and NPD

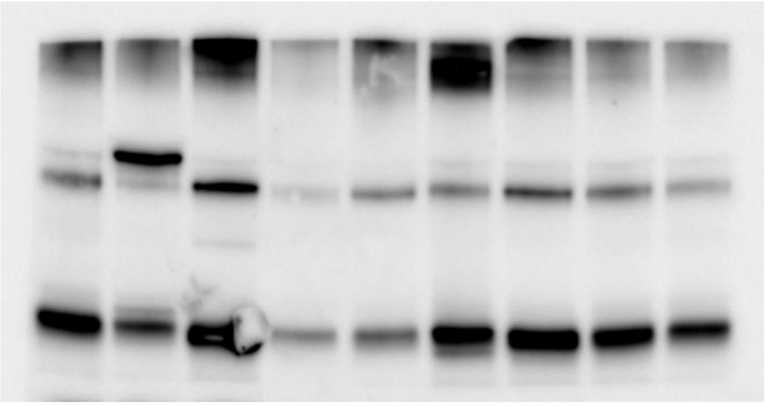

↑ ↑ ↑

PD I NPD C

Anti-λ: PD I and NPD

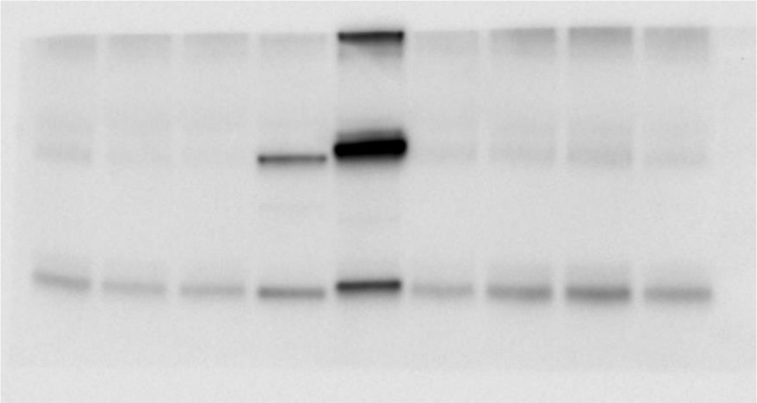

↑ ↑ ↑

PD I NPD C

Anti-κ: PD I1 (A and B: two different exposure times)

A

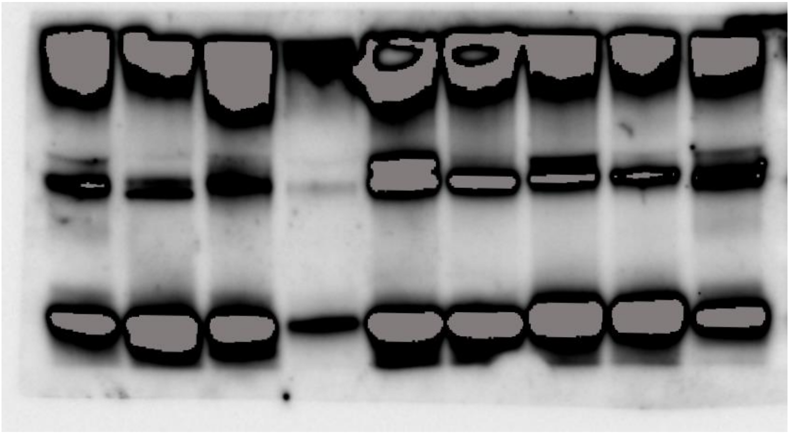

↑ ↑

PD II **C**

B

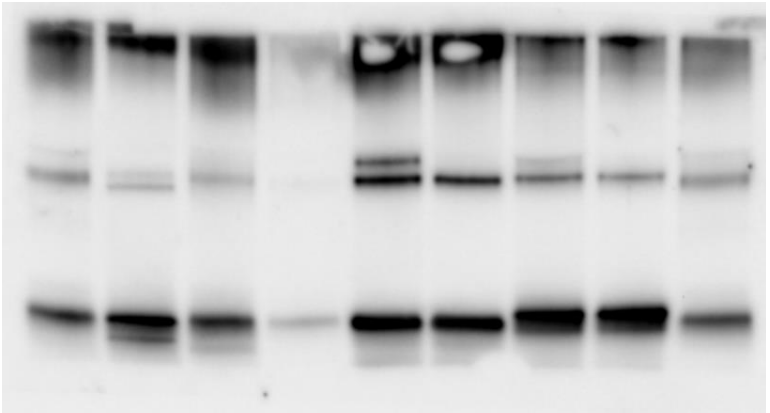

↑ ↑

PD II **C**

Anti-λ: PD II

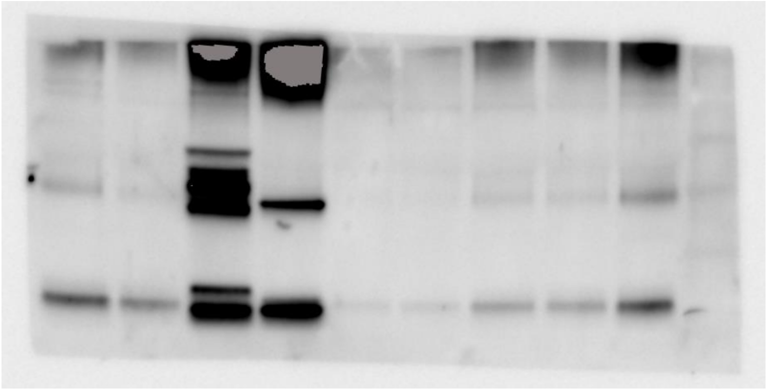

↑ PD II

↑ C

Anti-κ: PD III1 and IV

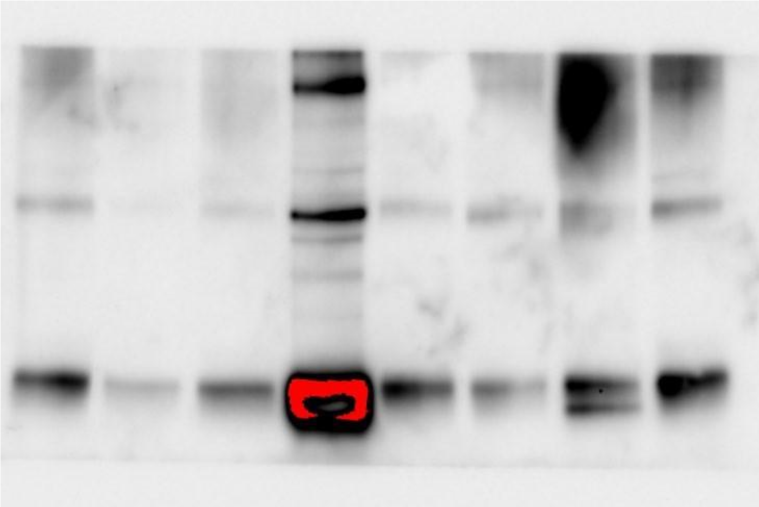

PD III PD IV C

Anti-λ: PD III and IV

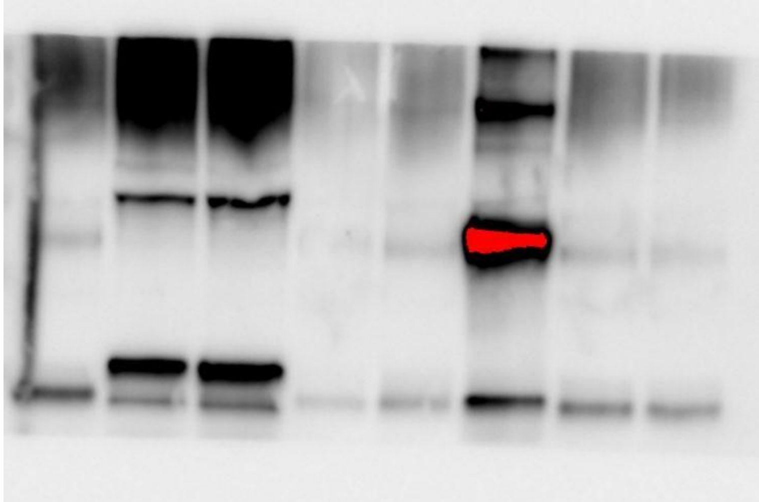

PD III PD IV C
